# Use of Bacterial Extracellular Vesicles for Gene Delivery to Host Cells

**DOI:** 10.3390/biom12091171

**Published:** 2022-08-24

**Authors:** Su-Im Kim, Jae Yeong Ha, Song-Yi Choi, Su-Hyung Hong, Heon-Jin Lee

**Affiliations:** 1 Department of Microbiology and Immunology, School of Dentistry, Kyungpook National University, Daegu 41940, Korea; 2 Craniofacial Nerve-Bone Network Research Center, Kyungpook National University, Daegu 41940, Korea

**Keywords:** extracellular vesicle, microRNA, vaccine, gene delivery

## Abstract

Extracellular vesicles (EVs), which are nanosized membranous particles secreted from both prokaryotic and eukaryotic cells, can deliver various biological molecules, such as nucleic acids, proteins, and lipids, into recipient cells. However, contrary to what is known about eukaryotic EVs, whether bacterial EVs (bEVs) can be used as transporters for bioactive molecules is becoming a hot area of research. In this study, we electroporated enhanced green fluorescent protein (EGFP) genes and precursor microRNA of Cel-miR-39 (pre-Cel-miR-39) from isolated bEVs of *Escherichia coli* and *Lactobacillus reuteri*. The EGFP plasmid, synthetic EGFP RNA, and pre-Cel-miR-39 were successfully delivered into the murine microglial BV2 cells via bEVs. PCR and confocal microscopy analysis confirmed the transfer of the EGFP plasmid and RNA. The bEV-delivered exogenous pre-Cel-miR-39 was further processed into the mature form of Cel-miR-39; its incorporation into Ago2—a major component of the RNA-induced silencing complex—was assessed using RNA-immunoprecipitation–PCR. Taken together, bEVs can be used as vehicles to deliver genetic materials and for novel biotechnological applications, such as gene transfer and mRNA vaccines.

## 1. Introduction

Delivery of biomolecules via nanocarrier-based systems has been extensively studied in regard to drug delivery and next-generation vaccine development [1]. Although the use of lipid- or polymer-based nanoparticles as a powerful and versatile delivery method for mRNA-based vaccines has been favored, the delivery agents contain ingredients that are capable of inducing allergic reactions and can therefore cause severe side effects in several cases [2]. Furthermore, extensive studies are being conducted on DNA-based vaccines, which are similar to mRNA vaccines; however, the viral vectors that are used as carriers may induce various side effects. Therefore, there is an emerging need for the rapid development of safe, novel vaccines and drug delivery systems.

Both gram-positive and gram-negative bacteria naturally secrete bacterial extracellular vesicles (bEVs), which are approximately 20–400 nm in size and contain various cargoes with biological activities, such as proteins, lipids, and genetic materials (DNA and RNA) [3,4]. Although endotoxins or liposaccharides (LPS) may be present in gram-negative bEVs, recent ongoing studies on the interaction between bEVs and immune functions have indicated the potential use of pathogen-producing intact and bioengineered bEVs as vaccines [5,6]. In this regard, both eukaryotic and prokaryotic EVs have gained great interest as therapeutic drug (including RNA molecules) vehicles for the treatment of certain cancer types, immune disorders, and other diseases [7,8,9]; however, information regarding the host application of ectopic (foreign) DNA or RNA transferred by bEVs for use in gene delivery is limited.

Using the reporter gene enhanced green fluorescent protein (EGFP), we demonstrated that exogenous electroporated DNA or RNA can be successfully delivered via bacterial EVs into the murine microglial cell line BV2. Furthermore, the electroporated precursor form of eukaryotic microRNA can be processed into the mature form of synthetic miRNA in BV2 cells and can be functionally activated when incorporated into the Ago2 protein. Taken together, it is possible to develop a new type of vaccine using EVs of commensal bacteria; further, bEVs have great potential as gene carriers in the development of gene therapy tools.

## 2. Materials and Methods

### 2.1. Culture

*Escherichia coli* (K12 strain) was grown in Luria-Bertani broth (BD Difco, Franklin Lakes, NJ, USA) at 37 °C until the optical density at 600 nm reached 0.5. *Lactobacillus reuteri* (ATCC 23272) was grown in de Man, Rogosa, and Sharpe broth (BD Difco) under anaerobic conditions.

### 2.2. Isolation and Analysis of bEVs

*E. coli* EVs were isolated from the concentrated supernatant using the ExoBacteria™ OMV Isolation Kit (SBI, Mountain View, CA, USA). *L. reuteri* EVs were isolated using gradient ultracentrifugation as previously described [10]. The isolated bEVs were treated with 1 μL of RNase A (1 U/μL, Thermo Fisher Scientific, Waltham, MA, USA) and DNase I (2 U/μL, Thermo Fisher Scientific) to make a final volume of 1.5 mL EV, followed by incubation at 37 °C for 25 min. Isolated bEVs were then analyzed using a NanoSight system (NanoSight NS300; Malvern Panalytical, Malvern, UK) to visualize the size distribution and quantity of bEVs.

### 2.3. Cell Viability Analysis after Treatment with bEVs

To investigate the effect of *E. coli* and *L. reuteri* EVs on cell viability, BV2 cells were seeded in 96-well plates (10,000 cells/well). The following day, the cells were treated with different concentrations of bEVs in fresh medium and incubated for another 24 h. Cell viability was then assessed using the MTT (3-[4,5-dimethyl-2-thiazolyl]-2,5-diphenyl-2H-tetrazolium bromide) assay, and the absorbance was read at 570 nm using a microplate reader. The results were obtained from three independent experiments; each bar represents the standard deviation.

### 2.4. Vector and RNA Preparation

The pGFP-N1 vector (Clontech, Mountain View, CA, USA) was used for EGFP plasmid DNA and in vitro EGFP transcription. The EGFP RNA was synthesized using the HeLaScribe Nuclear Extract in vitro Transcription System (Promega, Madison, WI, USA) with the plasmid gene (pGFP-N1) as a template, according to the manufacturer’s protocol.

### 2.5. Electroporation of Plasmid and RNA into bEVs

Plasmid (500 ng) and in vitro transcribed RNA (1 μg) were electroporated into bEVs (300 μL) under conditions of V = 150 and C = 100 μF. Immediately after electroporation, cells were transferred onto ice. The bEVs were treated with 1 μL of RNase A (1 U/μL, Thermo Fisher Scientific) and DNase I (2 U/μL, Thermo Fisher Scientific) to make a final volume of 1.5 mL; the mix was incubated at 37 °C for 25 min.

### 2.6. RNA Immunoprecipitation

For Ago2 RNA immunoprecipitation (RIP), BV2 cells were incubated with plasmids or RNA-containing bEVs. After 24 h, the culture media were removed, each plate was washed with 10 mL of phosphate-buffered saline (PBS), and the cells were collected and lysed using radioimmunoprecipitation assay lysis buffer (Thermo Fisher Scientific). Lysates (500 μg) were incubated with Dynabeads Protein G (Thermo Fisher Scientific) conjugated with 2 μL of Ago2-specific monoclonal antibody (2A8; Novus Biologicals, Littleton, CO, USA) or without the antibody. The beads were repeatedly washed with PBS, and TRIzol was added to extract bound RNA, which was then subjected to Cel-miR-39-3p (hereafter, Cel-miR-39) RIP–PCR.

### 2.7. Reverse Transcription-Quantitative PCR (RT-qPCR)

RT-PCR was performed to confirm the presence of the electroporated and transferred EGFP plasmid and RNA. The extracted RNA was used to synthesize cDNA using the Omniscript RT Kit (Qiagen, Valencia, CA, USA). For EGFP detection, the synthesized cDNA and positive control EGFP plasmid DNA were tested using PCR primers (EGFP primer sequence: EGFP-F: 5′-CTGGTCGAGCTGGACGGCGACG-3′; EGFP-R: 5′-CACGAACTCCAGCAGGACCATG-3′; 631 bp). Thereafter, electrophoretic separation on an 2% agarose gel plate was performed to examine the presence of the primer band and to verify the transformation of the plasmid gene. For Cel-miR-39 RIP–RT-qPCR, total RNA (15 ng) was reverse-transcribed using the TaqMan MicroRNA Reverse Transcription Kit (Applied Biosystems, Foster City, CA, USA). From the 20 μL reaction mixture, 1.33 μL was used for RT-qPCR, which was performed using the TaqMan Universal PCR Master Mix (Applied Biosystems) and TaqMan probes (Applied Biosystems).

### 2.8. Confocal Microscopy Analysis

Plasmid DNA or synthetic RNA-electroporated bEVs were added to BV2 cells cultured on chamber slides and incubated for 24 h at 37 °C. The slides were then washed three times with 1 mL of PBS, followed by incubation with DAPI (Vector Laboratories, Burlingame, CA, USA). The fluorescence signals were visualized using a laser scanning confocal microscope (LSM Zeiss 800; Carl Zeiss Microscopy, Jena, Germany).

### 2.9. Cel-miR-39 Precursor RNA Synthesis

Cel-miR-39 was chosen because it exists only in *Caenorhabditis elegans* and not in any other known mammalian cells. To obtain the RNA oligo of pre-Cel-miR-39, DNA oligo (5′-UAUACCGAGAGCCCAGCUGAUUUCGUCUUGGUAAUAAGCUCGUCAUUGAGAUUAUCACCGGGUGUAAAUCAGCUUGGCUCUGGUGUC-3′) along with the complementary strand of pre-miR-Cel-39 was synthesized by Bioneer (Daejeon, Korea). DNA oligos were annealed and cloned into the pGEM-T Easy vector (Promega), followed by in vitro transcription using the T7 promoter of the vector with an EZ T7 High Yield In Vitro Transcription Kit (Enzynomics, Daejeon, Korea). The correct clones with the pGEM-T easy vector were confirmed by sequencing after insertion and orientation verification by agarose gel electrophoresis.

### 2.10. Transmission Electron Microscopy (TEM)

The purified bEV samples were diluted 50 times with PBS and applied to 200-mesh Formvar/Carbon grids (Ted Pella, Inc., Redding, CA, USA). Samples were dried on the grids and viewed with a transmission electron microscope (HT7700, Hitachi, Tokyo, Japan) operated at 100 kV.

### 2.11. Statistical Analysis

All data are presented as mean ± standard deviation (SD). A significant variation analysis was used to calculate the parametric two-tailed unpaired *t*-test. All analyses were performed using Origin 8.0 (OriginLab, Northampton, MA, USA). *p*-values ≤ 0.05 were considered statistically significant. 

## 3. Results

### 3.1. bEV Analysis and BV2 Cell Viability Assay

**Figure 1:**
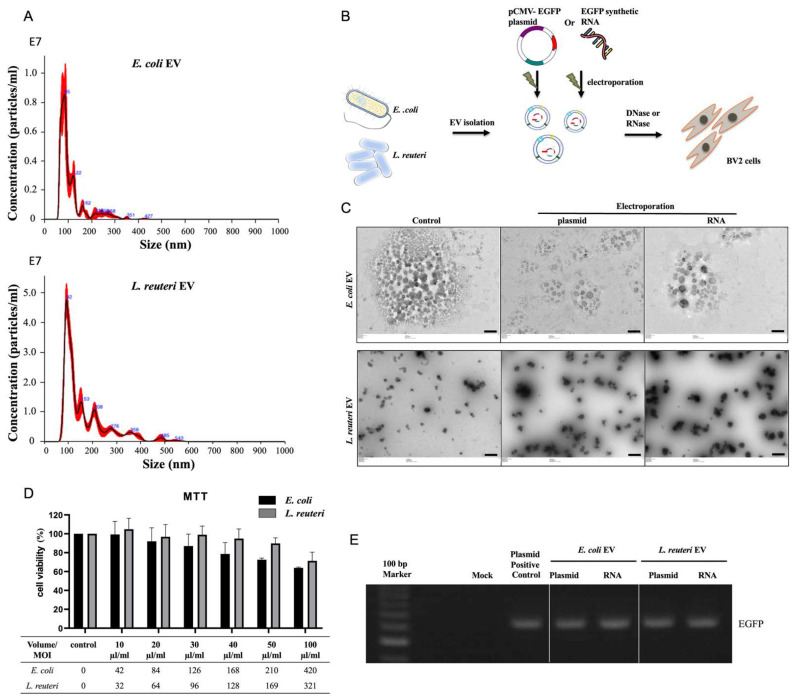

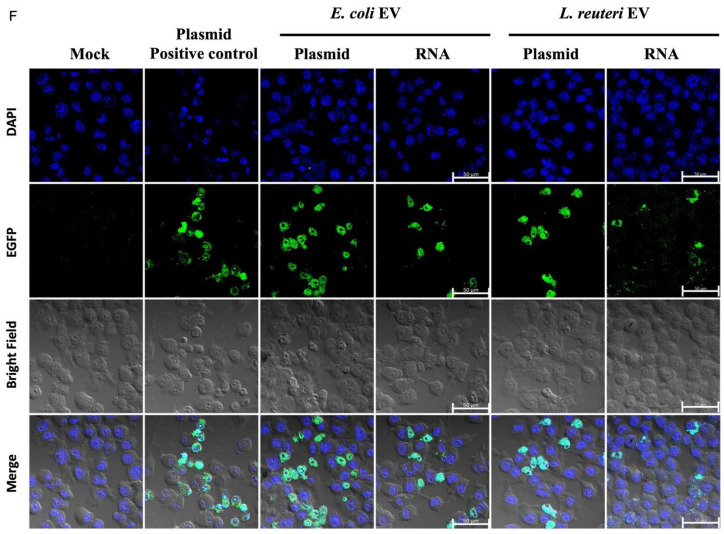
Bacterial extracellular vesicles (bEVs) can transfer exogenous plasmid or RNA to mammalian host cells (BV2). (**A**) Analysis of bEVs from *Escherichia coli* (top) and *Lactobacillus reuteri* (bottom) was performed using the NanoSight system to show the size distribution (*X*-axis) and particle numbers (*Y*-axis). (**B**) Schematic diagram of the experimental procedure. Enhanced green fluorescent protein (EGFP) plasmid or synthetic EGFP RNA was electroporated into EVs isolated from *E. coli* or *L. reuteri* followed by treatment with nucleases to remove the remaining plasmid DNA or RNA. (**C**) Transmission electron micrograph of bEVs. The images show small vesicles of each bacteria in comparison with bEVs after plasmid DNA or RNA electroporation. Scale bar, 300 nm. (**D**) Cell viability assay after treatment with bEVs. MTT assay was performed to investigate the effect of *E. coli* and *L. reuteri* EVs on cell viability. These results are from three independent experiments; each bar represents standard deviation. (**E**) RT-PCR was performed to verify plasmid DNA or synthetic EGFP RNA transferred by bEVs. (**F**) Delivery of EGFP DNA or RNA into BV2 cells. The bEVs were electroporated with the EGFP plasmid DNA or RNA. BV2 cells were incubated with *E. coli* (approximately 8.4 × 10^7^ particles; multiplicity of infection (MOI) of 84) and *L. reuteri* EVs (approximately 6.4 × 10^7^ particles; MOI of 64) on a chamber slide for 24 h at 37 °C. The cells were counterstained with DAPI (blue) to visualize the nuclei. Positive controls were transfected with EGFP plasmid using Lipofectamine 2000. Confocal microscopy analysis of bEVs reveals colocalized bEVs and EGFP (overlay). Scale bar = 50 μm.

Isolated bEVs were analyzed using the NanoSight system and TEM and were mostly 50–400 nm in size (Figure 1A,C). No visible differences were observed in bEVs following DNA or RNA electroporation (Figure 1C). bEVs can harm host cells by harboring toxic agents. The toxicity results showed that cell viability decreased by 80% after treatment with a multiplicity of infection (MOI) of 168 for *E. coli* and a MOI of 321 for *L. reuteri* (Figure 1D). Thereafter, we infected 1 × 10^6^ BV2 cells with *E. coli* and *L. reuteri* EVs at MOIs of 84 and 64, respectively, for avoiding the toxic effect of bEVs under our experimental conditions.

### 3.2. Transfer of EGFP DNA and RNA via bEVs

The delivery of EGFP DNA or RNA into BV2 cells was verified using RT-PCR and confocal fluorescence microscopy. The bEVs were electroporated with EGFP plasmid DNA or RNA and were treated with DNase or RNase A to remove the free unloaded nucleic acids (Figure 1B). RT-PCR was performed to verify the delivery of plasmid DNA or synthetic EGFP RNA carried by bEVs, and the PCR product was of the expected size (631 bp, Figure 1E). Confocal microscopy analysis of the bEVs showed EGFP gene expression in BV2 cells after treatment with the EGFP plasmid or RNA-containing bEVs (Figure 1F).

### 3.3. Exogenous Maturation of Pre-Cel-miR-39 and Incorporation into Ago2 in the Host Cells via bEVs

**Figure 2:**
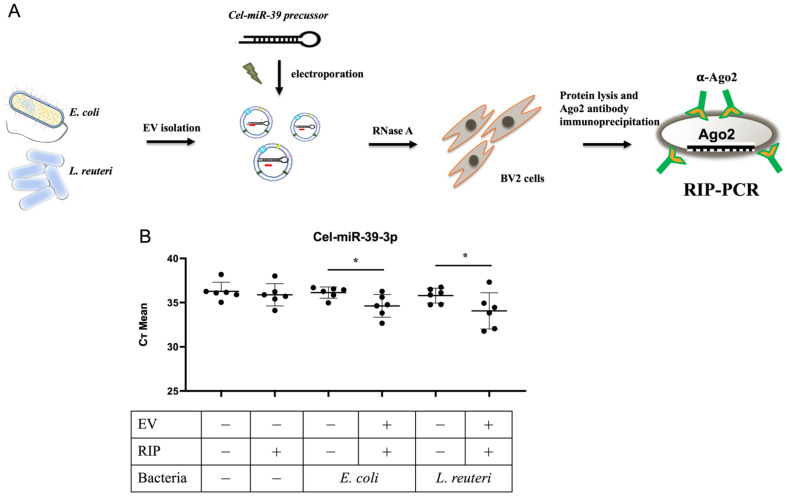
Exogenous pre-Cel-miR-39 via bacterial extracellular vesicles (bEVs) can be transformed into their mature form and incorporated into Ago2 in the host cells. (**A**) Schematic diagram of the experimental procedure. Cel-miR-39 precursor RNA was electroporated into EVs isolated from *Escherichia coli* and *Lactobacillus reuteri* and then treated with RNase A to remove the remaining RNA. BV2 cells were then treated with Cel-miR-39 electroporated bEVs and lysed for further incubation with anti-Ago 2 antibody. (**B**) RNA immunoprecipitation (RIP)- PCR was performed to detect the mature form of Cel-miR-39 that was bound to Ago2 protein using TaqMan specific Cel-miR-39 probe. The lower Ct values indicate higher Cel-miR-39 expression (error bars indicate standard deviation). Ct values higher than 36 (in control samples) indicated absence of Cel-miR-39. * *p* ≤ 0.05.

To investigate whether the precursor miRNA delivered by bEVs can mature in the host cells, RT-qPCR was performed to detect the matured form of Cel-miR-39 bound to Ago2 protein using a TaqMan specific Cel-miR-39 probe after treatment with pre-Cel-miR-39 containing bEVs (Figure 2A).

Regarding detection of matured form of Cel-miR-39, lower Ct values indicated higher Cel-miR-39 expression (Figure 2B; six biological samples; error bars indicate SD). Ct values higher than 36 (shown in control samples) indicated the absence of Cel-miR-39. Ct values of pre-Cel-miR39 containing bEV were noticeably lower than those of the controls, suggesting that exogenous miRNA precursors can be processed into their mature form in the host cells and be successfully incorporated into the Ago2 protein via bEVs.

## 4. Discussion

Although endogenous miRNA-sized small RNAs in bEVs can be transferred to host cells [11,12], whether exogenous RNA or DNA can be introduced into bEVs and delivered to host cells remains largely unknown. However, substantial research has shown that eukaryotic EVs (exosomes) can be genetically altered to carry miRNAs and inhibitors to recipient cells [13,14].

Our results suggest that bEVs can be used as vehicles for nucleic acids. DNA or RNA cargo can be introduced into bEVs by electroporation, which are capable of successfully transferring their nucleic acid cargo into mammalian host cells in vitro. In this study, both the DNA and RNA forms of EGFP were delivered into the host cells to produce EGFP proteins. Furthermore, precursor miRNA can also be activated to its mature form by binding to the Ago2 protein, which is a major component of the RNA-induced silencing complex and results in the inhibitory function of miRNAs [15]. These results strongly imply that transferring exogenous DNA or RNA via bEVs into host cells sustains the activity of the nucleic acids, as they are not degraded or removed by the host immune system. Our findings also indicate that *L. reuteri* EVs are less hazardous (Figure 1D), possibly because of the absence of LPS, and that more particles can be utilized for host infection.

The bEVs can transfer their contents horizontally into cells of other species. For example, almost 40 years ago, it was reported that *Haemophilus influenza* secreted DNA via EVs to protect DNA during transformation [16]. More recently, microbial RNA and protein cargo in bEVs have been systemically delivered into host cells in an animal model [17]. Other studies have also presented evidence regarding host–microbe interkingdom transfer of extracellular RNAs via bEVs [18,19].

Based on our previous studies wherein we found that bEVs and extracellular RNAs (exRNAs) can cross the blood–brain barrier of mice [11,12], we hypothesize that bEVs can be used to treat various neuronal diseases and for developing mRNA vaccines. Recently, mRNA-based vaccines have been developed using different systems based on high-efficiency, non-toxic RNA carriers that can prolong antigen expression. In addition, novel adjuvants have been developed for greater immune responses [20]. bEVs may therefore comprise a practical vaccine platform for delivering mRNA or DNA to induce host immune responses. Furthermore, as bEVs contain bacterial substances, they can be used as natural adjuvants for such vaccines. That bEVs can be easily and quickly purified industrially is also advantageous.

Further in-depth studies are needed, including the fine-tuning of necessary bEV particle numbers and conditions for infection. There exists great potential for using engineered bEVs as genetic tools for successful drug delivery as well as novel vaccine development.

## Data Availability

Not applicable.
